# Interpretation and management of AGC cytology

**DOI:** 10.1186/1471-2334-14-S7-P41

**Published:** 2014-10-15

**Authors:** Cristina Vasiliu, Simona Elena Albu, Cătălina Alexandrescu, Diana Mihai

**Affiliations:** 1 Carol Davila University of Medicine and Pharmacy, Bucharest, Romania; 2 Department of Obstetrics and Gynecology, University Emergency Hospital of Bucharest, Romania

## Background

Cervical cytology has continually evolved. Nowadays, the category of atypical glandular cells (AGC) is less frequent than atypical squamous cells (ASC), associating poorly-reproducible inter-observer interpretation. Cellular characters sometimes surpass benign reparatory processes or associate high-grade squamous precursor lesions, glandular neoplasia, including cervical adenocarcinoma, endometrial neoplasm and even extrauterine adenocarcinomas. Therefore, AGC needs special attention and aggressive diagnostic procedures.

Nevertheless, the most common viral infection of the reproductive tract, the human-papillomavirus (HPV) infection, shouldn’t be underestimated, as about 30% of AGC cases are HPV-positive. The presence of HPV in AGC patients identifies a group at higher-risk for developing cervical neoplasia. Studies show that 70% of AGC-HPV-negative patients have no evidence of cervical cancer, and 76% of AGC-HPV-positive results may present glandular or squamous neoplasia.

## Methods

This is a retrospective study to determine the value of the AGC Pap test category in detecting endocervical and endometrial pathology and the potential pitfalls of false positive diagnosis. It was performed in the University Emergency Hospital of Bucharest during March/2009 – December/2013. The total number of presentations for cervical pathology was 14,219. The diagnosis of AGC was found in 58 cases (0.41%).

## Results

The age range was 24 to 59y (median age 41).

In 46 cases (79%) AGC was the only cytologic anomaly found. In 12 cases the AGC cytology was associated with squamous lesion: ASC-US (6 cases), LSIL (1 case), ASC-H (1 case) and HSIL (4 cases).

Most colposcopies (58%) were negative for lesions, some were non-satisfactory (12%), 5 (8.6%) revealed polyps, 12 (20%) had squamous lesions; there was no colposcopic diagnosis of adenocarcinoma.

Among the 58 cases of AGC 17 (29.3%) were HPV-positive.

Follow-up histology in 42 cases revealed: cervical or endometrial hyperplasia, metaplasia or polyps, endocervical or endometrial adenocarcinoma, cervical squamous carcinoma, CIN 1/2/3, endocervical leiomyoma, herpetic infection, inflammation, endometrial atrophy, endometriosic ovarian cyst. There were 12 cases with negative pathology findings and 16 cases without biopsy.

## Conclusion

There is a large panel of pathology, other than glandular, or no pathology at all, responsible for AGC cytology.

AGC encompasses endometrial glandular neoplasia in addition to cervical glandular neoplasia and squamous neoplasia and, less commonly, metastatic disease from more distant sites.

There is an association between HPV infection and the gravity of endocervical lesions; patients who test positive should be carefully followed.

There is an association between age and diagnosis. Young patients are likely to have squamous lesions while older ones are likely to have glandular neoplasia.

